# Impact of individual metabolic risk components or its clustering on endothelial and smooth muscle cell function in men

**DOI:** 10.1186/s12933-016-0394-5

**Published:** 2016-05-17

**Authors:** Michio Shimabukuro, Namio Higa, Hiroaki Masuzaki, Masataka Sata, Shinichiro Ueda

**Affiliations:** Department of Cardio-Diabetes Medicine, Institute of Biomedical Sciences, Tokushima University Graduate School, 3-18-15 Kuramoto, Tokushima, 770-8503 Japan; Cardiovascular Department, Naha City Hospital, Okinawa, Japan; Division of Endocrinology, Diabetes and Metabolism, Hematology, Rheumatology (Second Department of Internal Medicine), Graduate School of Medicine, University of the Ryukyus, Okinawa, Japan; Department of Cardiovascular Medicine, Institute of Biomedical Sciences, Tokushima University Graduate School, Tokushima, Japan; Department of Clinical Pharmacology and Therapeutics, Graduate School of Medicine, University of the Ryukyus, Okinawa, Japan

**Keywords:** Obesity, Metabolic syndrome, Endothelial function, Insulin resistance

## Abstract

**Background:**

Impaired vasoreactivity is often observed in subjects with metabolic syndrome, a condition that includes the presence of a specific cluster of risk factors for obesity and cardiovascular disease. However, hierarchical causes in the impaired vasoreactivity have not been clarified. We evaluated the impact of individual metabolic risk components or its clustering under the condition of insulin resistance on endothelial and smooth muscle cell function.

**Methods:**

Vascular reactivity to acetylcholine (Ach), with or without nitric oxide synthase (NOS) inhibitor *N*^G^-monomethyl-l-arginine (L-NMMA), or sodium nitroprusside (SNP) by forearm venous occlusion plethysmography and insulin sensitivity index (*M* mg/kg/min) in euglycemic clamp were measured in men without (n = 18, control group) or with (n = 19, metabolic syndrome group) metabolic syndrome.

**Results:**

(1) Ach-induced maximal forearm blood flow (maxFBF) was impaired in subjects with metabolic syndrome. In particular, the NOS-dependent component of Ach-induced maxFBF was selectively decreased, while the NOS-independent component remained relatively unchanged. (2) Ach-induced maxFBF and ∆Ach-induced maxFBF with L-NMMA were correlated with waist circumference, glucose, and triglycerides, and most strongly correlated with visceral fat area, adiponectin, and *M*. (3) Multivariate regression analysis indicated that individual metabolic risk components explained Ach-induced maxFBF by 4–21 %. Clustering of all metabolic risk components increased this to 35 %, and the presence of metabolic syndrome explained 30 %, indicating that defining metabolic syndrome can effectively predict impairment of endothelial dysfunction.

**Conclusions:**

Endothelial dysfunction was correlated with individual metabolic risk components, but more strongly with clustering of the components under a condition with low insulin sensitivity. We suggest that in subjects with metabolic syndrome, endothelial function is impaired by multiple cardiovascular risk factors exclusively when under the condition of insulin insensitivity and also that defining metabolic syndrome can effectively predict impairment of endothelial dysfunction.

## Background

Metabolic syndrome is a condition that includes the presence of a specific cluster of risk factors for obesity and cardiovascular disease, including abdominal obesity, high blood pressure, impaired fasting blood glucose, hypertriglyceridemia and low HDL cholesterol [1]. Of note, the clinical utility of metabolic syndrome has been questioned [2, 3], because different definitions and different clusterings of components of metabolic syndrome may result in variations in cardiovascular risk predictions [4].

Impairment of vasoreactivity has been observed in patients with traditional coronary risk factors, even in the absence of morphological atherosclerotic lesions [5]. Accordingly, the assessment of vasoreactivity can provide pivotal information as a diagnostic and prognostic tool in patients at risk for atherosclerotic cardiovascular disease [6, 7]. The vasoreactivity is often impaired in subjects with metabolic syndrome [8, 9]. Hence, the impaired vasoreactivity in this syndrome can be provoked by two scenarios [1]: (1) individual metabolic risk components such as high blood pressure, hyperglycemia, and dyslipidemia, or (2) its clustering under the condition of obesity and/or insulin resistance. However, the principal scenario for the vascular dysfunction in metabolic syndrome remains obscure.

The current study compared the impact of individual metabolic risk components and its clustering under the condition of obesity and/or insulin resistance on endothelial and smooth muscle cell function in subjects with metabolic syndrome.

## Methods

### Subjects

Male subjects were divided into either the group without metabolic syndrome (n = 18, control group) or with metabolic syndrome (n = 19, MS group). A subject was defined as having metabolic syndrome as per the guidelines outlined in the IDF consensus statement [10]. Therefore, the subject had metabolic syndrome if he was obese (according to the Japanese criteria, having a waist circumference ≥85 cm in men) and had any two of the following four factors: (1) hypertriglyceridemia [serum triglyceride concentration ≥150 mg/dL (1.69 mmol/L)], (2) a low HDL cholesterol level [serum HDL cholesterol concentration of 40 mg/dL (1.04 mmol/L)], (3) an elevated blood pressure (systolic blood pressure ≥130 mmHg and/or diastolic blood pressure ≥85 mmHg) or was taking anti-hypertensive drugs, and (4) a high fasting plasma glucose level [fasting plasma glucose concentration ≥100 mg/dL (5.6 mmol/L)]. Participants who were taking insulin regimen or oral anti-diabetic drugs were excluded. Waist circumference was measured in the standing position and subcutaneous fat area (SFA) and intra-abdominal visceral fat area (VFA) were determined at the level of the umbilicus using a standardized method involving computed tomography [11]. Subjects were instructed to refrain from vigorous exercise, anti-hypertensive drugs, anti-hyperlipidemic drugs, non-steroidal anti-inflammatory drugs, alcohol, smoking and caffeine for 24 h prior to the study day. The study protocol was approved by the Ethical Committee of the University of the Ryukyus, and obeyed to the standards set by the Declaration of Helsinki. Written informed consent was obtained from all subjects.

### Biochemical measurements

Venous blood samples were obtained in tubes containing EDTA-sodium (1 mg/mL) and in polystyrene tubes without an anticoagulant. The EDTA-containing tubes were promptly chilled. Plasma was immediately separated by centrifugation at 3000 rpm and 4 °C for 10 min, and serum isolated by centrifugation at 1000 rpm at room temperature for 10 min. Samples were stored at −80 °C until they were assayed. Routine chemical methods were used to determine the serum concentrations of total cholesterol, HDL cholesterol, triglycerides, creatinine, glucose, and electrolytes. The serum concentration of LDL cholesterol was estimated using Friedewald’s method [12].

### Euglycemic hyperinsulinemic clamp

The whole-body insulin sensitivity index (*M*) was measured using a hyperinsulinemic euglycemic clamp [13] with modifications [14] for 180 min. A primed continuous infusion of insulin (10.8 pmol kg^−1^ min^−1^, Novo-Nordisk, Japan) was administered along with a variable rate infusion of 20 % dextrose (Baxter Health Care, Japan) that was adjusted manually to maintain serum glucose of 5.2 mmol/L. This was determined based on arterialized samples withdrawn every 5 min from an ipsilateral right dorsal hand vein (heated-air blanket was kept at 55 °C). The *M* value (mg/kg/min) was calculated during the last 30 min of the study.

### Vascular reactivity

Forearm blood flow (FBF) was measured simultaneously in both forearms by bilateral venous occlusion plethysmography with mercury-in-silicone elastomer strain gauges, as described [14]. All subjects were supine in a quiet, air-conditioned room, with both forearms resting slightly above their heart level. Acetylcholine (Ach, 0–400 nmol/min) with or without *N*^G^-monomethyl-l-arginine (L-NMMA, 8 µmol/min), a nitric oxide synthase (NOS) inhibitor, or sodium nitroprusside (SNP, 0–30 nmol/min) was infused into a 27-gauge catheter inserted into the brachial artery of the non-dominant arm by volumetric precision pumps. The infusion rate was maintained at 1 mL/min throughout the study unless otherwise indicated. All FBF data were obtained via a Mac Lab Version 4 chart recorder (AD Instruments, Hamstead, London, United Kingdom).

### Statistical analysis

Values are expressed as the mean ± SD. Comparisons of vascular responses were analyzed by two-way analysis of variance (ANOVA) for repeated measures on one factor, followed by Holm–Sidak multiple comparison test to compare group means. Simple regression analysis was used to identify significant linear associations of vascular responses with components of the metabolic syndrome and related variables. Multiple regression analysis for vascular reactivity was performed in standard sequential (hierarchical) models and in a stepwise backward model using variables of individual components in the definition of metabolic syndrome and markers for possible underlying mechanisms. All analyses were performed using Jump version 12.1.0 software (SAS Institute Inc., Cary, NC). A *P* value of <0.05 was considered statistically significant.

## Results

### General characteristics

Subject characteristics are summarized in Table 1. All subjects with or without metabolic syndrome completed the study. Subjects in the MS group were clinically obese, and displayed a higher body weight, BMI, and waist circumference than subjects without metabolic syndrome. The MS group also had greater SFA and VFA. There were no significant differences in age, heart rate, or smoking status (control 20 %, MS 16 %) between the two groups. The MS group exhibited higher levels of fasting glucose, insulin, HbA1_c_, total and LDL cholesterol, and triglycerides and lower levels of HDL cholesterol than the control group. The MS group exhibited lower adiponectin than the control group (p < 0.0001). In addition, *M* values were reduced by half in the MS group.

**Table 1:** General characteristics of the studied patients

	Control (n = 18)	Metabolic syndrome (n = 19)
Age (years)	48 ± 15	51 ± 9
Body weight (kg)	65.3 ± 8.4	76.4 ± 8.3**
Body mass index (kg/m^2^)	23.1 ± 2.3	27.8 ± 2.7**
Waist circumference (cm)	84.1 ± 9.8	93.9 ± 7.2**
Systolic blood pressure (mm Hg)	121 ± 13	134 ± 19**
Diastolic blood pressure (mm Hg)	74 ± 7	81 ± 10*
Heart rate (beats/min)	68 ± 8	68 ± 11
Glucose (mmol/L)	5.7 ± 1.6	7.5 ± 2.7*
Insulin (pmol/L)	44.5 ± 28.9	79.0 ± 42.0**
Haemoglobin A1c (%)	5.4 ± 1.1	7.0 ± 2.1*
LDL cholesterol (mmol/L)	2.79 ± 0.70	3.13 ± 0.66
HDL cholesterol (mmol/L)	1.41 ± 0.48	1.22 ± 0.20
Triglyceride (mmol/L)	1.12 ± 0.70	2.53 ± 1.04**
Adiponectin (log µg/ml)	0.87 ± 0.15	0.72 ± 0.12**
*M* (mg/kg/min)	7.81 ± 2.86	4.14 ± 2.04**

Mean ± SD, * p < 0.05, ** p < 0.01 vs control

### Vascular reactivity

#### Vascular responsiveness to Ach

**Fig. 1:**
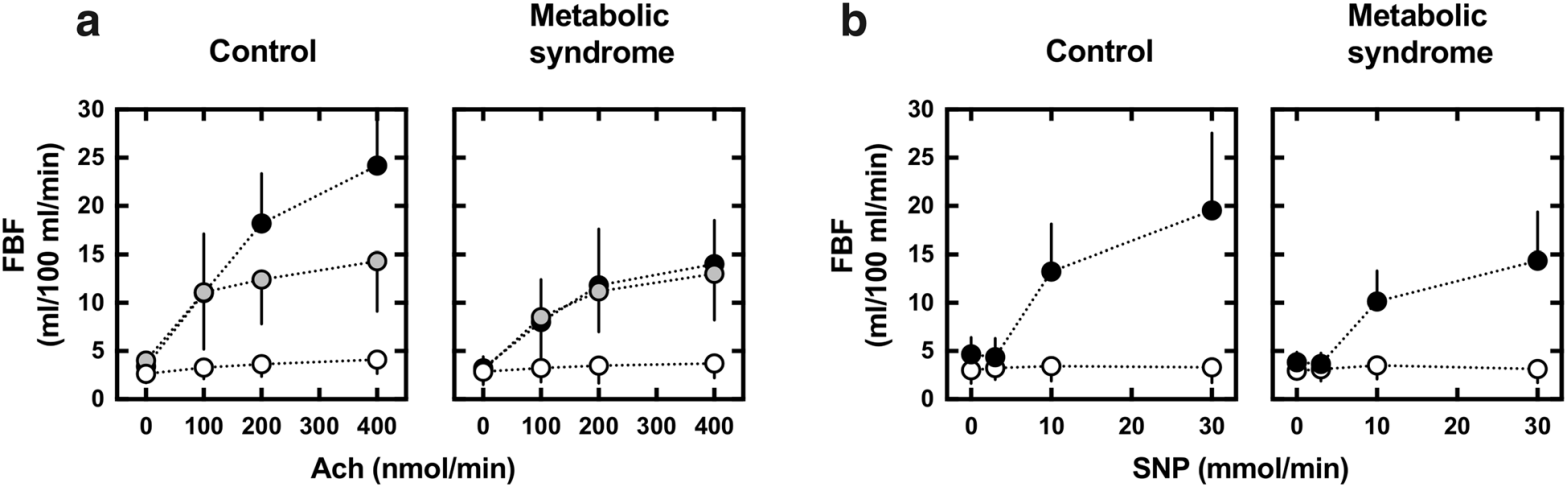
Forearm vascular reactivity to **a** ACh, with or without the nitric oxide synthase (NOS) inhibitor L-NMMA (8 μmol/min), or **b** SNP, as measured by bilateral venous occlusion plethysmography in subjects without (n = 18) or with (n = 19) metabolic syndrome. FBF was measured simultaneously in subjects infused with Ach (*closed circles*), SNP (*closed circles*) or Ach plus L-NMMA (*gray circles*) and non-infused arms (*open circles*) using bilateral venous occlusion plethysmography. *FBF* forearm blood flow, *ACh* acetylcholine, *L-NMMA* *N* ^G^-monomethyl-l-arginine, *SNP* sodium nitroprusside. Data are expressed as mean ± SD

Basal FBF was 3.4 ± 1.3 mL/min/100 mL in the control group and 3.2 ± 1.2 mL/min/100 mL in the MS group (p = 0.663). As shown in Fig. 1a, Ach-induced maximal FBF (Ach-induced maxFBF) (•) was reduced in the MS group (14.0 ± 4.5 mL/min/100 mL) compared to the control group (24.2 ± 8.0 mL/min/100 mL, p < 0.0001).

#### NOS-dependent vasodilation

Ach-induced maximal FBF during co-infusion of L-NMMA was reduced to 14.3 ± 5.2 mL/min/100 mL in the control group, but was not reduced in the MS group (13.0 ± 4.8 mL/min/100 mL) (Fig. 1a). The decline in Ach-induced maximal FBF by L-NMMA (∆maxFBF by L-NMMA), which represents the NOS-dependent vasodilation, was 10.2 ± 6.7 and 2.1 ± 2.4 mL/min/100 mL, respectively (p < 0.0001).

#### Smooth muscle responsiveness to SNP

As shown in Fig. 1b, SNP-induced maximal FBF (SNP-induced maxFBF) was also reduced in the MS group compared to control group: the SNP-induced maxFBF was 19.5 ± 8.0 mL/min/100 mL in the control group and 14.4 ± 5.0 mL/min/100 mL in the MS group (p = 0.021).

### Vascular reactivity and components of metabolic syndrome

**Fig. 2:**
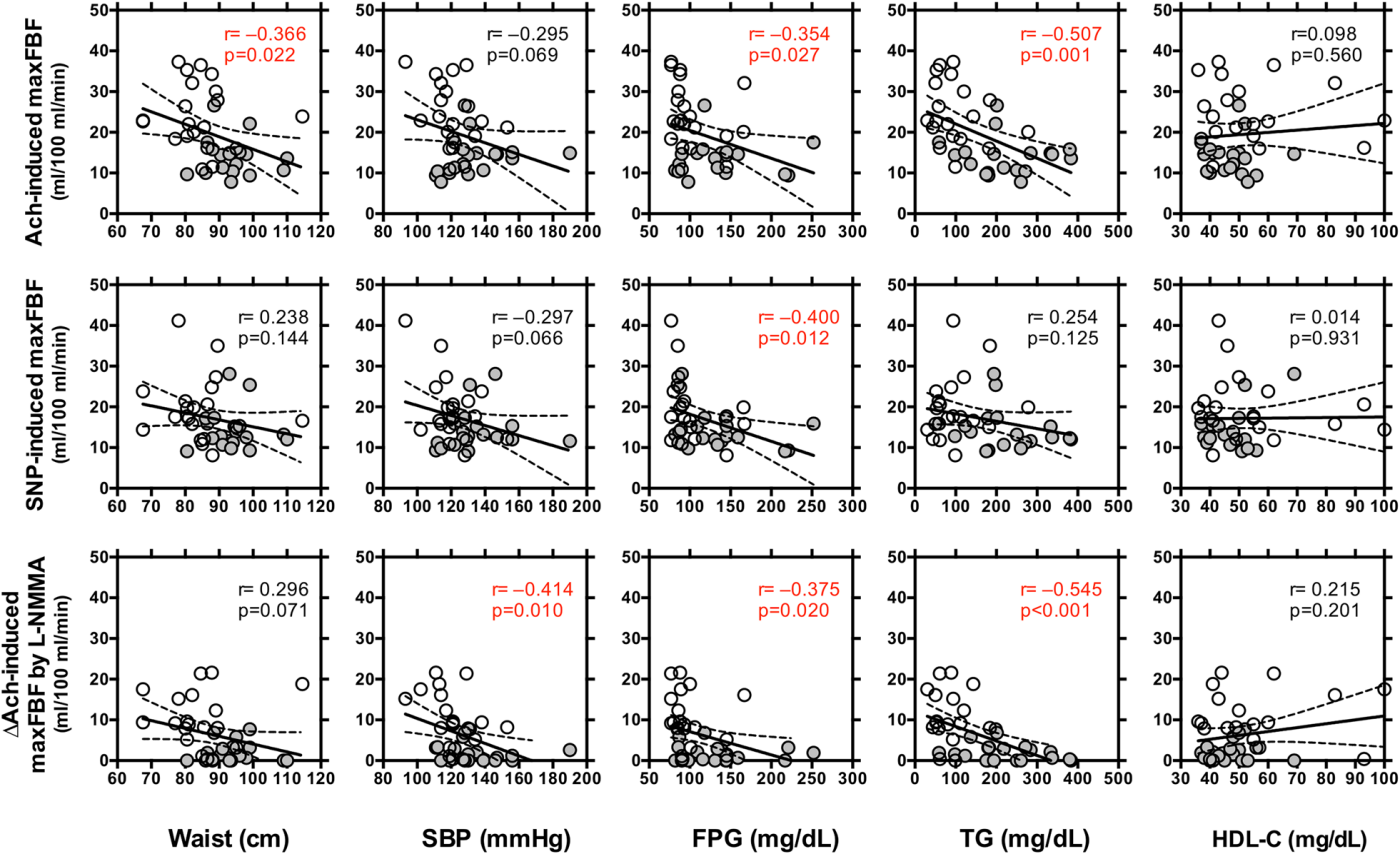
Simple regression analysis between vascular reactivity and components of metabolic syndrome in men without (n = 18, *open circles*) or with (n = 19, *gray circles*) metabolic syndrome. FBF at an Ach infusion of 400 nmol/min, FBF at an SNP infusion of 30 nmol/min, and ∆Ach-induced maximal FBF by co-infusion of L-NMMA at 8 μmol/min. Linear regression analysis was done, and r and p values are shown

To explore the contributions of individual metabolic risk components to altered vascular control, the relationships between the components and endothelial and vascular smooth muscle responses were assessed. Significant relationships were observed between Ach-induced maxFBF and waist circumference, glucose, and triglycerides, such that higher levels of these components were associated with lower vasodilation (Fig. 2). SNP-induced maxFBF was correlated only with glucose. In addition, ∆maxFBF by L-NMMA was correlated with SBP, glucose and triglycerides.

### Vascular reactivity, abdominal fat distribution, adiponectin, and insulin sensitivity

**Fig. 3:**
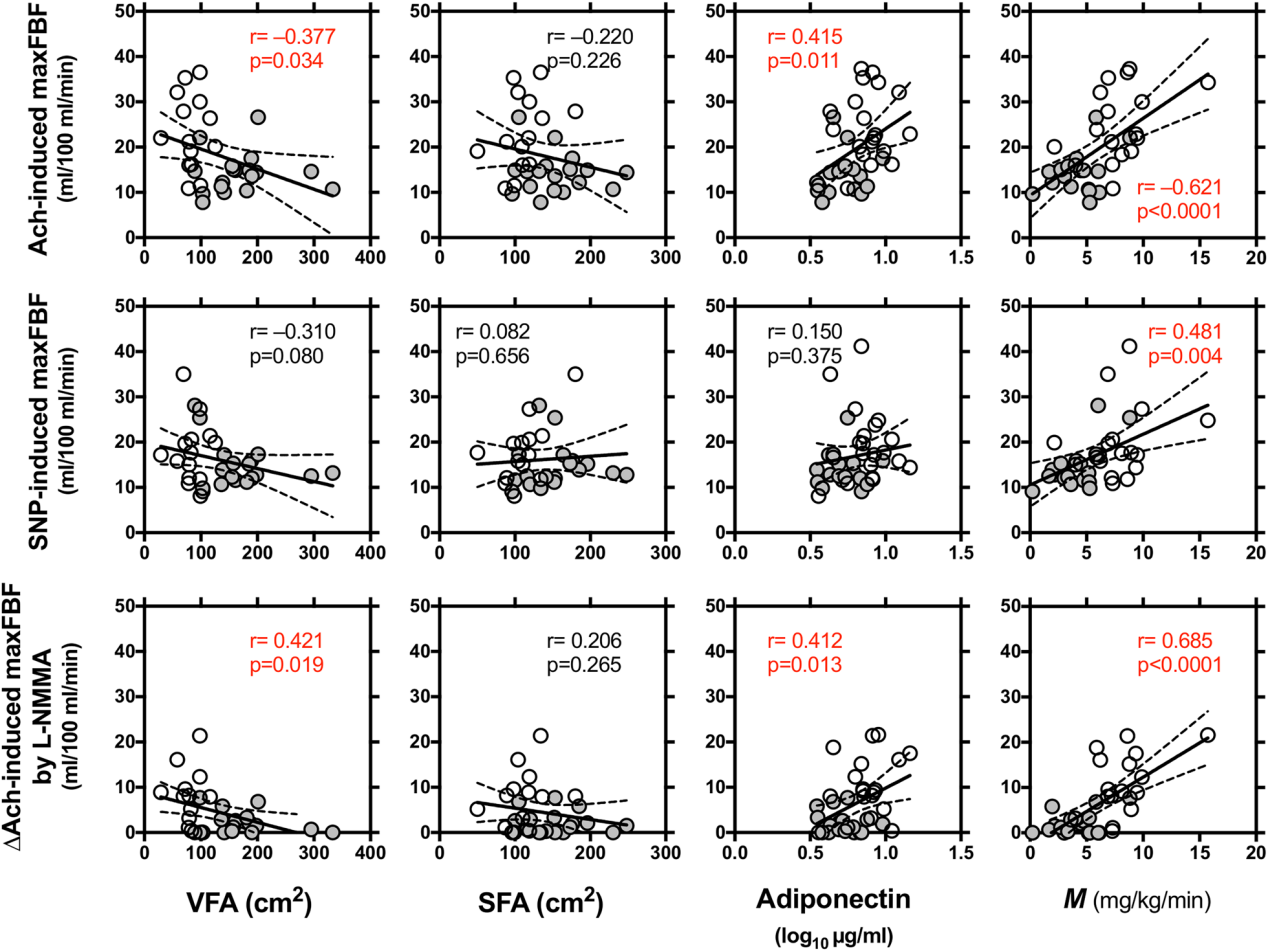
Simple regression analysis between vascular reactivity, abdominal fat distribution, adiponectin and insulin sensitivity index (*M*) in men without (n = 18, *open circles*) or with (n = 19, *gray circles*) metabolic syndrome. FBF at an Ach infusion of 400 nmol/min, FBF at an SNP infusion of 30 nmol/min, and ∆Ach-induced maximal FBF by co-infusion of L-NMMA at 8 μmol/min. Pearson’s correlation coefficients and p values are shown

Next, we evaluated the impact of VFA, SFA, adiponectin, and *M* value, the markers for possible underlying mechanisms, on Ach-induced maxFBF (Fig. 3). A strong negative relationship was observed between Ach-induced maxFBF and VFA, but not SFA. Ach-induced maxFBF was positively correlated with adiponectin, and most strongly correlated with *M* value. SNP-induced maximal FBF was not correlated with VFA, SFA, or adiponectin, but was positively correlated with *M* value. The ∆maxFBF by L-NMMA was correlated with SBP, glucose and triglycerides. In addition to Ach-induced maxFBF, ∆maxFBF by L-NMMA was negatively correlated with VFA, and positively correlated with adiponectin and *M* value.

### Multivariate regression analysis of vascular reactivity determinants

Next, we determined the impact of individual metabolic risk components or the clustering of components on vascular reactivity by multivariate regression models (Table 2).

**Table 2:** Multivariate regression analysis fo vascular reactivity

	Model 1^a^		Model 2^a^		Model 3^a^		Model 4^a^		Model 5^a^		Model 6^b^	
*Ach-induced maximal forearm blood flow (FBF)*												
Corrected R^2^	0.228		0.347		0.514		0.297		0.429		0.522	
P value	0.009		0.006		0.001		0.002		0.000		0.000	
Variables	E	P	E	P	E	P	E	P	E	P	E	P
Age (years)	−0.153	0.156	−0.018	0.868	0.085	0.427	−0.109	0.288	0.016	0.883	0	0.712
Current smoking (yes or no)	1.246	0.336	0.532	0.665	−0.770	0.517	0.188	0.876	−0.991	0.419	0	0.562
Waist circumference (cm) ≥85 cm (yes or no)	4.749	0.002	3.387	0.024	2.137	0.116					0	0.134
SBP ≥130 or DBP ≥85 mmHg or use of antihypertensive drugs			1.931	0.146	2.613	0.046					0	0.326
Glucose ≥100 mg/dL (yes or no)			1.947	0.131	−0.374	0.771					0	0.184
Triglycerides ≥150 mg/dL (yes or no)			2.230	0.095	1.305	0.298					0	0.490
HDL-cholesterol ≥150 mg/dL (yes or no)			1.065	0.456	1.493	0.286					0	0.965
Metabolic syndrome (yes or no)							4.675	0.000	−3.120	0.022	−3.560	0.006
*M* (mg/kg/min)					1.424	0.005			1.258	0.012	1.186	0.005
*SNP-induced maximal forearm blood flow (FBF)*												
Corrected R^2^	0.123		0.117		0.148		0.173		0.224		0.254	
P value	0.063		0.159		0.152		0.026		0.022		0.0017	
Variables	E	P	E	P	E	P	E	P	E	P		
Age (years)	−0.233	0.020	−0.153	0.168	−0.114	0.346	−0.213	0.029	−0.151	0.162	0	0.240
Current smoking (yes or no)	1.239	0.293	0.875	0.474	0.229	0.864	0.848	0.448	0.232	0.848	0	0.906
Waist circumference (cm) ≥85 cm (yes or no)	1.669	0.202	1.477	0.304	0.507	0.735					0	0.818
SBP ≥130 or DBP ≥85 mmHg or use of antihypertensive drugs			0.437	0.736	1.123	0.432					0	0.415
Glucose ≥100 mg/dL (yes or no)			2.355	0.069	0.649	0.655					0	0.438
Triglycerides ≥150 mg/dL (yes or no)			−0.091	0.944	−0.810	0.563					0	0.726
HDL-cholesterol ≥150 mg/dL (yes or no)			0.792	0.576	1.492	0.343					0	0.519
Metabolic syndrome (yes or no)							−2.120	0.061	−1.022	0.430	0	0.336
*M* (mg/kg/min)					0.872	0.107			0.784	0.103	1.237	0.002
*∆Ach-induced maximal forearm blood flow (FBF) by L-NMMA*												
Corrected R^2^	0.236		0.460		0.594		0.406		0.533		0.481	
P value	0.009		0.001		0.000		0.000		<0.0001		<0.0001	
Variables	E	P	E	P	E	P	E	P	E	P	E	P
Age (years)	−0.179	0.029	−0.071	0.344	−0.013	0.866	−0.145	0.045	0.074	0.415	0	0.094
Current smoking (yes or no)	0.635	0.512	−0.007	0.993	−0.726	0.397	−0.069	0.935	0.854	0.407	0	0.558
Waist circumference (cm) ≥85 cm (yes or no)	3.048	0.008	1.975	0.068	1.112	0.265					0	0.918
SBP ≥130 or DBP ≥85 mmHg or use of antihypertensive drugs			1.874	0.049	2.161	0.028					0	0.216
Glucose ≥100 mg/dL (yes or no)			1.541	0.085	0.141	0.877					0	0.594
Triglycerides ≥150 mg/dL (yes or no)			1.731	0.073	1.403	0.131					0	0.360
HDL-cholesterol ≥150 mg/dL (yes or no)			1.673	0.100	1.856	0.075					0	0.774
Metabolic syndrome (yes or no)							−3.627	0.000	0.950	0.008	−15.01	0.000
*M* (mg/kg/min)					0.867	0.015			0.341	0.021	0	0.112

^a^Model 1–5: standard multiple regression analysis

^b^Model 6: stepwise multiple regression analysis

For Ach-induced maxFBF, waist circumference (model 1) and triglycerides, but not blood pressure, glucose or HDL cholesterol (data not shown), were determinants of vascular reactivity, even after correcting for age and smoking status. Clustering of 5 components increased the corrected R^2^ (model 2), and addition of *M* value to the clustering of 5 components further increased the corrected R^2^, which reached 0.514 (model 3). Meanwhile, the presence of metabolic syndrome, though slightly decreased compared to the clustering of 5 components, was also a determinant of Ach-induced maxFBF (model 4); addition of *M* value to the presence of metabolic syndrome increased the corrected R^2^ (model 5).

For SNP FBF, waist circumference (model 1), blood pressure, plasma glucose, triglycerides, and HDL cholesterol (data not shown), were not determinants of vascular reactivity. Clustering of these components with or without *M* value (models 2 and 3), and the presence of metabolic syndrome with or without *M* value (models 4 and 5) were also not determinants of vascular reactivity.

For ∆maxFBF by L-NMMA, waist circumference (model 1) and triglycerides, but not blood pressure, glucose or HDL cholesterol (data not shown), were determinants of vascular reactivity. Clustering of 5 components and the presence of metabolic syndrome increased the corrected R^2^ value (models 2 and 3), and addition of *M* value further increased the corrected R^2^ value (models 4 and 5).

In the stepwise backward model (model 6) including the above individual components, defining metabolic syndrome and *M* value were significant predictors of Ach-induced maxFBF, *M* value for SNP-induced maxFBF, and defining metabolic syndrome for ∆maxFBF by L-NMMA.

### Vascular reactivity and the number of components of metabolic syndrome

**Fig. 4:**
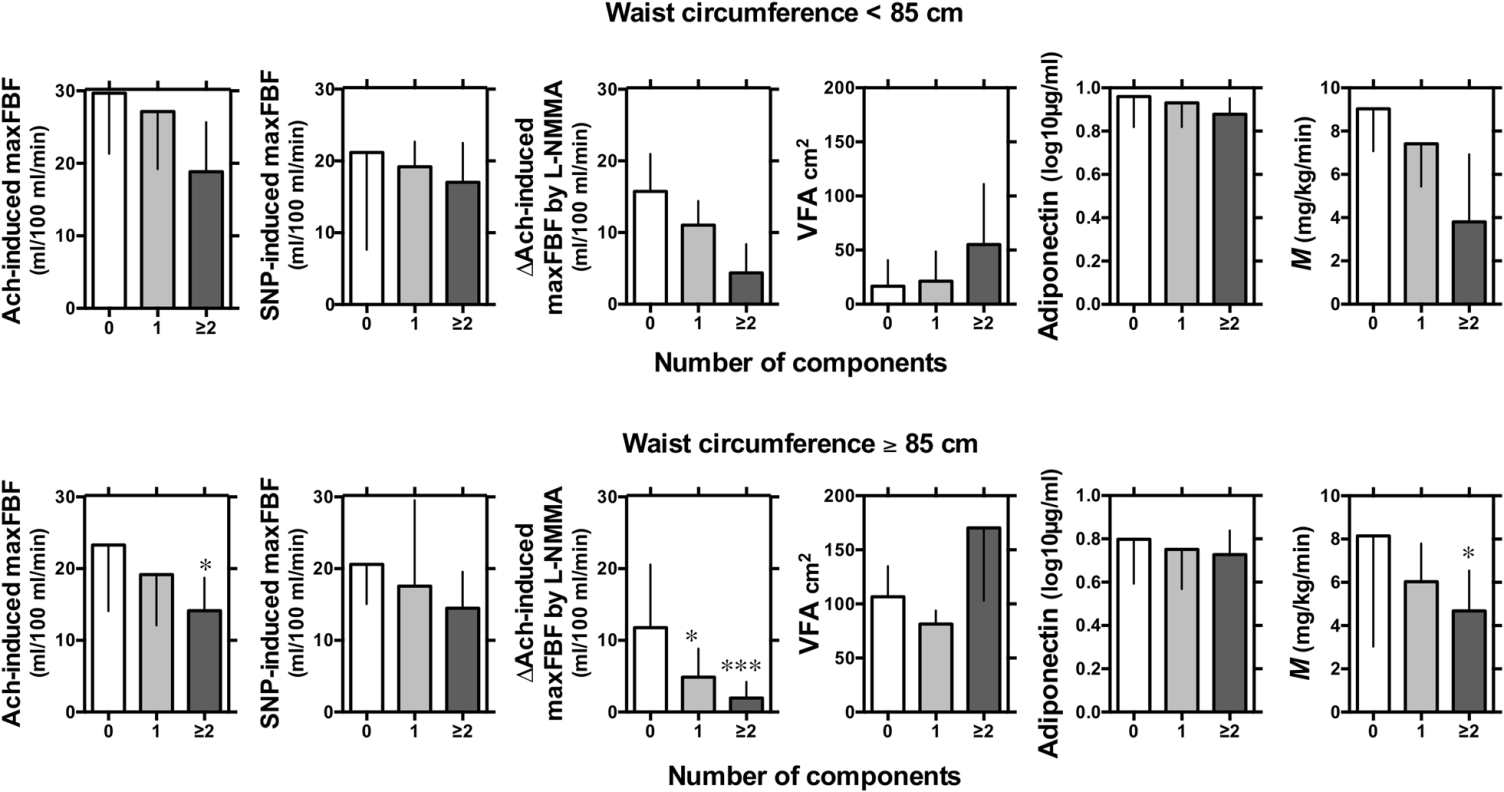
The relationship between vascular reactivity and number of components of metabolic syndrome in men without visceral obesity (n = 12, waist circumference <85 cm) or with visceral obesity (n = 25, waist circumference ≥85 cm). Subjects were divided into groups with 0, 1 or 2 ≥of the following metabolic syndrome components: high level of fasting glucose, elevated blood pressure, hypertriglyceridemia, and low level of HDL cholesterol. Data are expressed as mean ± SD. Statistical analysis was done by two-way analysis of variance (ANOVA), followed by Holm–Sidak multiple comparison test to compare group means. *P* values = * <0.05, *** <0.001 vs. the 0 group

Finally, we evaluated the relationship between vascular reactivity and the number of metabolic risk components in subjects with or without visceral obesity (Fig. 4). In subjects without visceral obesity (waist circumference <85 cm), subjects with 1 or ≥2 of 4 components, which included a high level of fasting glucose, elevated blood pressure, hypertriglyceridemia, and a low level of HDL cholesterol, did not show a statistical difference in Ach-induced maxFBF, SNP and ∆maxFBF by L-NMMA, compared to the group with 0 component. There were no significant differences in VFA, adiponectin and *M* value among the subgroups with 0, 1 and ≥2 components. In subjects with visceral obesity (waist circumference ≥85 cm), Ach-induced maxFBF was not decreased in the group with 1 component compared to the group with 0 component, but was decreased in the group with ≥2 components. SNP was not different among the subgroups with 0, 1 and ≥2 components. The ∆maxFBF by L-NMMA was decreased in accordance with the number of the components. In the group with ≥3 components, there were no significant differences in VFA and adiponectin, but *M* value was significantly decreased.

## Discussion

The new findings of this study are: (1) Ach-induced and SNP-induced maximal FBFs were impaired in subjects with metabolic syndrome. In particular, we first found that the NOS-dependent component of Ach-induced maxFBF was selectively decreased, while the NOS-independent component remained unchanged. (2) Ach-induced maximal FBF and ∆Ach-induced maximal FBF by L-NMMA, and SNP-induced maximal FBF were correlated strongly with *M* value than with individual metabolic components. (3) Multivariate regression models clearly indicated that defining metabolic syndrome, as compared to individual metabolic components, predicts impairment of endothelial dysfunction.

### Vascular reactivity

As observed in previous studies of obese subjects [15, 16], Ach-induced maxFBF, a marker of endothelial function, was reduced in MS group. We further investigated vascular NO bioavailability by comparing the dose–response curves of Ach with and without pharmacological NOS inhibition. In the NO clamp technique, the NOS-dependent and NOS-independent components of FBF can be accurately determined [17]. Co-infusion of L-NMMA and Ach caused a decrease in the level of inhibition of Ach-induced maxFBF in subjects with metabolic syndrome, and equalized the Ach-induced maximum FBF during NOS inhibition between the two groups. This implies that the NOS-dependent component of Ach-induced maxFBF was selectively decreased in subjects with metabolic syndrome, while the NOS-independent component remained relatively unchanged.

Classically, it has been believed that endothelial function is impaired, even in the first step of atherosclerosis, but smooth muscle cell function is preserved even in the advanced stages of atherosclerosis [5]. Conversely, current study showed that SNP-induced maximal FBF, a marker of smooth muscle responsiveness, was reduced in the MS group. The data obtained during NO inhibition by L-NMMA, suggest that the whole difference in Ach reactivity between two groups can be mainly due to a defect in the NOS-dependent NO synthesis. However, the impaired smooth muscle vasorelaxation suggests an impaired bioavailability and/or responsiveness to endogenous NO [18]. Given that the response to Ach was superimposable at the levels in NO synthesis and/or its biological activities, the contradictory findings may be partially explained by a converse compensation for the loss in NO bioavailability [18]. Endothelium-derived hyperpolarizing factor (EDHF) pathway, in which alterations was reported under pathological conditions, might be one such candidate [19]. Fernandes et al. reported that time-to-peak after hyperemia rather than flow mediated dilation (FMD) distinguished metabolic syndrome from healthy controls [20]. In the time-course analysis of FMD [21], time to peak after hyperemia is not influenced by L-NMMA inhibition, suggesting that other factors, such as differences in vascular compliance and transduction independently of the NOS pathway [20, 22].

### Vascular reactivity, abdominal fat distribution, adiponectin and insulin sensitivity

In the current study, Ach-induced maximal FBF, and ∆Ach-induced maximal FBF by L-NMMA were correlated with waist circumference, glucose and triglycerides, and more strongly with *M* value. This result agrees with a previous study [15] where Steinberg et al. report that obesity/insulin resistance is associated with both blunted endothelium-dependent vasodilation and failure of euglycemic hyperinsulinemia to augment endothelium-dependent vasodilation. This suggests that obese/insulin-resistant subjects are characterized by endothelial dysfunction and endothelial resistance to insulin’s effect on enhancement of endothelium-dependent vasodilation. Our results further indicated that each of abdominal fat distribution, adiponectin, and insulin sensitivity was correlated with Ach-induced maximal FBF and ∆Ach-induced maximal FBF by L-NMMA more greatly than each of the MS components. Therefore, endothelial function in metabolic syndrome can be explained mostly by mutually dependent insulin resistance, visceral obesity and hypoadiponectinemia through increased production of reactive oxidative species (ROS) and proinflammatory cytokines [11, 23–29].

Impairment in the SNP-induced maximal FBF was also correlated with *M* value as well as in Ach-induced maximal FBF. The observation may be supported by Schinzari et al. showing that the vascular responsiveness to both Ach and SNP was not enhanced during hyperinsulinemia in patients with metabolic syndrome [30]. They suggests that insulin’s facilitator action on the vasodilator machinery was caused by endothelium unresponsiveness (a decrease in NO synthesis and/or a loss of its biological activities) and by defective sensitization of smooth muscle vasorelaxation (a loss in NO bioavailability) [19]. Aoqui et al. found distinct patterns of microvascular dysfunction in metabolic syndrome, with augmented vasoconstriction present in the initial phase of metabolic syndrome independent of endothelial dysfunction [31]. Reportedly, locally produced ROS [25] and/or fat-derived ROS [23] can react with NO, generate peroxynitrite, and impair cyclic GMP-dependent vasodilatation; this mechanism may partially explain these smooth muscle cell dysfunction [18, 31].

### Impact of individual metabolic risk components and its clustering on endothelial and smooth muscle cell function

Since metabolic syndrome is a cluster of relatively heterogeneous atherosclerotic risk factors, which may solely impair endothelial function, we evaluated the relationship between vascular reactivity and individual components or its clustering. In a simple regression model (Fig. 2), the Ach-induced maxFBF and ∆maxFBF by L-NMMA were lineally correlated with waist circumference, glucose and triglycerides. However, multivariate regression analysis indicated that individual components explained only partially Ach-induced maxFBF by 4–21 %, ∆maxFBF by L-NMMA by 6–31 %, and SNP-induced maxFBF by 8–17 % (data not shown). In model 3, including the 5 components of visceral obesity, high fasting glucose, elevated blood pressure, hypertriglyceridemia, and a low level of HDL cholesterol, increased the assumption to 35 % in Ach-induced maxFBF and 46 % in ∆maxFBF by L-NMMA, but only 16 % in SNP-induced maxFBF. These results support the notion that clustering of the components more greatly estimates endothelial dysfunction than individual components, but not smooth muscle cell function. Notably, the presence of metabolic syndrome, while still slightly less than the clustering of 5 components (model 2), explained 30 % of Ach-induced maxFBF and 41 % of ∆maxFBF by L-NMMA (model 4). These findings support the notion that defining metabolic syndrome is effective in predicting endothelial dysfunction, which can subsequently predict future cardiovascular events [5, 7]. In the current study, type 2 diabetes mellitus was prevalent in the MS group (12/18, 67 %) than in the control group (4/19, 21 %) (p < 0.05). When all patients divided into diabetic and non-diabetic groups, Ach-induced maxFBF (21.5 ± 1.7 vs 16.0 ± 2.0 mL/min/100 mL, p = 0.040), ∆maxFBF by L-NMMA (8.3 ± 6.8 vs 2.8 ± 4.2 mL/min/100 mL, p = 0.008) and SNP-induced maxFBF (19.4 ± 1.4 vs 13.6 ± 1.7 mL/min/100 mL, p = 0.011) were all impaired in diabetic groups. Vascular function in type 2 diabetes mellitus is also confounded by cardiovascular risk factors, such as obesity, hypertension and dyslipidemia, suggesting significance of cardiovascular risk clustering [32].

In our study, endothelial function is not strongly correlated with individual metabolic risk components, but strongly with insulin resistance. Thus, it is assumed that recovery of vascular function can be obtained less effectively by improvement of individual metabolic parameters [33], but more effectively by improvement of metabolic syndrome or insulin resistance [34]. This notion may be supported by the fact in patients with metabolically healthy obesity (MHO), a medical condition characterized by obesity which does not produce metabolic complications such as dyslipidemia, impaired glucose tolerance or hypertension [35]. The MHO had abnormal vascular reactivity, although their endothelial dysfunction was less pronounced than in patients with metabolic syndrome, indicating that obesity is associated with vascular damage independent of those metabolic abnormalities underlying metabolic syndrome [36].

We also evaluated the relationship between vascular reactivity and the number of metabolic syndrome components (Fig. 4). In subjects without visceral obesity, there were no differences in Ach-induced maxFBF and ∆maxFBF by L-NMMA among patients with 0, 1 and 2≥ of the 4 selected components. In contrast, in subjects with visceral obesity, Ach-induced maxFBF and ∆maxFBF by L-NMMA decreased in subjects with ≥2 components. As such, one or two components cannot be sufficient to cause impairment of endothelial function even in subjects with visceral obesity, but the clustering of ≥2 components can be sufficient. Interestingly, subjects without visceral obesity that have ≥2 components showed a subtler endothelial dysfunction than subjects with visceral obesity. This notion agrees with the previous study of Li et al., where they showed impaired endothelial function in subjects with metabolic syndrome as compared to individuals with a similar burden of traditional cardiovascular risk factors, but without metabolic syndrome [37]. Their multivariate regression model found that after adjustment for covariates and 6 traditional cardiovascular risk factors, the presence of metabolic syndrome had a significant and independent influence on endothelial function (p < 0.01).

In our multivariate model, addition of *M* value increased the corrected R^2^ for Ach-induced maxFBF to 51 % in the model including all the components (model 3) and to 43 % in the model including the presence of metabolic syndrome (model 5). Insulin resistance should play a pivotal role in causing endothelial dysfunction by comorbidity of the metabolic syndrome components. We also found that endothelial function, indicated by Ach-induced maxFBF and ∆maxFBF by L-NMMA, was impaired in the group with ≥2 components, and *M* value decreased according to the number of components. Collectively, our data suggest that in subjects with visceral fat obesity, endothelial function is impaired by multiple cardiovascular risk factors exclusively when under the condition of insulin insensitivity.

### Study limitations

First, we obtained data from a small number of subjects; therefore, there was the risk of type II errors. The strain gauge plethysmography and the euglycemic hyperinsulinemic clamp techniques, used in the study for measurement of vascular function and insulin sensitivity, are sensitive and solid, but the time-consuming and invasive characteristics limits number of participants. In contrast, alternative simplified methods such as flow-mediated dilation (FMD) [38] and HOMA-IR [39] are good for recruiting participants, but the reliability is limited. Thus, current results should be confirmed by combinations of multiple techniques and clinical studies with different size. Second, this cross-sectional study has shown only a correlation between vascular dysfunction and insulin resistance in subjects with metabolic syndrome, and has not indicated a cause-effect relationship. In future studies, we need to confirm that a therapeutic approach to improve insulin sensitivity by such as reductions in visceral fat obesity and ectopic fat deposition can recover vascular dysfunction in metabolic syndrome. Third, since metabolic syndrome is a cluster of relatively heterogeneous atherosclerotic risk factors, which solely affect endothelial function, careful consideration should be taken to evaluate underlying mechanisms. Forth, we could not determine the molecular mechanisms by which insulin resistance occurs and impairs endothelial function from the current study.

## Conclusions

The current study evaluated the impact of individual metabolic risk components or its clustering on endothelial and smooth muscle cell function in subjects with metabolic syndrome. The endothelial and smooth muscle cell function were correlated more strongly with clustering of the components under a condition with low insulin sensitivity. Therefore, it may be suggested that in subjects with metabolic syndrome, vascular reactivity is impaired by multiple cardiovascular risk factors exclusively under the condition of insulin insensitivity and also that defining metabolic syndrome can effectively predict impairment of vascular reactivity.
